# Burnout and quality of life among healthcare workers in central Uganda

**DOI:** 10.1371/journal.pone.0305713

**Published:** 2024-08-19

**Authors:** Amir Kabunga, Eustes Kigongo, Ponsiano Okalo, Samson Udho, Anna Auma Grace, Raymond Tumwesigye, Anne Ruth Akello, Marvin Musinguzi, Walter Acup, Jannat Nabaziwa, Enos Mwirotsi Shikanga, Haliama Namata

**Affiliations:** 1 Department of Psychiatry, Lira University, Lira, Uganda; 2 Department of Environmental Health and Disease Control, Lira University, Lira, Uganda; 3 Department of Midwifery, Lira University, Lira, Uganda; 4 Department of Community Health, Lira University, Lira, Uganda; 5 Department of Education Psychology, Moi University, Eldoret, Kenya; 6 Department of Mental Health, Makerere University, Kampala, Uganda; Lira University, UGANDA

## Abstract

**Background:**

The widespread problem of burnout among healthcare workers is not only common but also a significant concern that impacts the entire healthcare system in Uganda. It is essential to understand the connection between burnout and quality of life among healthcare workers in the specific context of central Uganda, where healthcare professionals face high patient volumes, limited resources, exposure to infectious diseases, and socioeconomic challenges. This study examined the relationship between burnout and quality of life among healthcare workers in central Uganda.

**Methods:**

This research utilized a cross-sectional study conducted across various healthcare settings in central Uganda. The data were analyzed at descriptive, bivariate, and multivariate levels. The relationship between dependent and independent variables was evaluated using an independent t-test for binary variables and a one-way analysis of variance (ANOVA) for categorical variables. Significance was determined with a reported p-value, with relationships deemed significant at p < 0.2. For multivariable analysis, multiple linear regression was employed using a forward selection method, with significance set at 5% (p < 0.05).

**Results:**

Our findings indicate that nearly 40% of healthcare workers reported experiencing high levels of burnout. The average score for overall quality of life was 10.71 (±4.89), with variations observed across different domains. The study reveals a significant connection between socio-demographic factors, burnout, and overall quality of life, emphasizing the impact of job category, supervisory support, sleep quality, and burnout on the well-being of healthcare workers. Predictive analysis illustrates how these factors influence both overall quality of life scores and scores in specific domains. Particularly noteworthy is that nurses and technicians tend to have a lower quality of life compared to physicians.

**Conclusion:**

The results underscore the relationship between socio-demographic factors, burnout, and particular aspects of quality of life. Notably, job category, supervisory support, sleep quality, and burnout stand out as significant factors shaping the well-being of healthcare workers. Nurses and technicians encounter distinct challenges, suggesting the need for interventions tailored to their needs. Addressing issues such as inadequate supervisory support, burnout, and sleep-related problems is recognized as a potential approach to improving the overall quality of life among healthcare workers.

## Introduction

The healthcare sector plays a crucial role in safeguarding the health of any society by preventing diseases and promoting public health. Healthcare workers in Africa, like those in many other regions, encounter numerous challenges that impact both their personal and professional lives [1]. Among these challenges, burnout stands out as a pressing issue [1]. Burnout is a complex psychological syndrome arising from prolonged workplace stress, and it has been identified as a significant concern in the healthcare sector [2]. Research has shown that burnout can have adverse effects on various aspects of an individual’s life, including decreased job satisfaction, impaired job performance, and increased turnover rates among healthcare professionals [3].

Burnout is a condition of extreme exhaustion resulting from prolonged stress, characterized by three key aspects: emotional exhaustion, depersonalization, and reduced personal accomplishment [4]. The definition of burnout has been updated in the latest edition of the International Classification of Diseases-11, framing it as a workplace issue encompassing these three syndromes [5], rather than solely a consequence of challenges in managing general life situations. Burnout typically manifests as excessive strain on both physical and emotional levels, feelings of cynicism and detachment towards one’s work, and a diminished sense of professional efficacy [6]. Research into burnout is essential as studies have shown that burnout syndrome among healthcare workers adversely affects patient outcomes [7]. Factors contributing to burnout include low job satisfaction, heavy workloads, job-related stress, lack of social support, female gender, and age [8, 9]. Burnout not only impacts job performance but also significantly affects the overall quality of life of healthcare workers, increasing the risk of experiencing poor wellbeing [10].

The World Health Organization defines quality of life as “the individual’s perception of their position in life in the context of the culture and value systems in which they live and in relation to their goals, experiences, standards, and concerns” [11]. Quality of life, a multidimensional concept encompassing physical, mental, and social well-being, is intricately linked to the professional experiences of healthcare workers [12]. In addition to biological outcomes, quality of life has been frequently used to measure wellbeing in health research [13]. However, while it has been extensively used among patients, there is a paucity of research on the quality of life among healthcare workers, especially in low-income countries. Additionally, studies on the relationship between quality of life and burnout among healthcare workers are also scarce. The available literature indicates that quality of life is low among healthcare workers with high levels of burnout [14, 15].

Burnout significantly impacts the overall well-being and quality of life of healthcare workers [16]. Burnout among healthcare workers manifests in various ways, including emotional exhaustion, depersonalization, and reduced personal accomplishment [17]. These factors collectively contribute to diminished job satisfaction and a sense of disillusionment with one’s career, which directly affects the quality of life. Healthcare workers often face challenges such as heavy workloads, inadequate staffing, and limited access to resources and support systems [18]. These factors exacerbate stress levels and contribute to feelings of exhaustion and frustration [19]. Furthermore, burnout can impact the physical health of healthcare workers, leading to increased risk of chronic conditions such as hypertension, cardiovascular disease, and musculoskeletal disorders [20]. This not only compromises their ability to deliver quality care but also undermines their own health and well-being, further diminishing their overall quality of life.

The pervasive issue of burnout among healthcare workers is not only prevalent but also a critical concern that affects the entire healthcare system in Uganda obstacles [9, 21]. It is crucial to comprehend the relationship between burnout and the quality of life among healthcare workers in the particular setting of central Uganda, where healthcare resources encounter high patient loads, insufficient resources, exposure to infectious infections, restricted training opportunities, and socioeconomic obstacles [9, 21]. The goal of this study was to investigate burnout among healthcare workers in central Uganda and how it affects their overall quality of life. The study offers insightful information that may help shape focused interventions and policies that will lessen burnout and improve the general health of healthcare workers in central Uganda. This will ultimately enhance the overall effectiveness and sustainability of healthcare delivery in central Uganda.

## Methods

### Study design

The present study employed a cross-sectional study in health facilities central Uganda between May and July 2023.

### Setting

The research was carried out in various healthcare facilities, both public and private, throughout central Uganda. Central Uganda, situated in the heart of East Africa, features a blend of urban and rural environments and includes the capital city, Kampala. Uganda’s healthcare system includes both public and private facilities, serving a diverse population across regions. Public facilities, typically government-operated, are essential in providing services ranging from tertiary hospitals to grassroots health centers. Private healthcare facilities, such as hospitals, clinics, and specialized centers, supplement the public sector, adapting to factors like urbanization and population density. Central Uganda plays a crucial role in the healthcare landscape, exhibiting a mix of urban and rural dynamics in facility distribution. Urban centers offer more hospitals and specialized services, while rural areas often depend on health centers and community clinics. The healthcare system faces challenges such as limited resources, understaffing, and infrastructure constraints, all of which are predictors of burnout in the region [22].

### Participants

The participants included physicians, nurses, and technicians who actively engaged in direct patient care and played integral roles in the health system in central Uganda. We ensured the representation of healthcare workers from both urban and rural healthcare settings within central Uganda. We employed a comprehensive sampling strategy targeting diverse healthcare facilities in the region, including both urban and rural areas. Urban facilities were chosen to capture experiences in metropolitan settings, while rural health centers ensured representation from remote communities facing unique challenges. Participant eligibility included actively practicing physicians, nurses, and technicians engaged in direct patient care in central Uganda’s health system. Exclusion criteria comprised individuals on extended leave and those with less than one year of experience to assess burnout’s impact on quality of life effectively.

### Sampling procedure and sample size

A cross-sectional study design was used to determine the sample size. To achieve a maximum sample size with a 95% confidence interval (CI) and a 5% margin of error, the proportion (P) was estimated to be 50%. Additionally, we increased the sample size to 550 healthcare workers to account for a 30% nonresponse rate and practical considerations. The 30% nonresponse rate in our study reflects the challenges of conducting research in real-world healthcare settings. Factors such as demanding work conditions, limited time for research, and logistical constraints contributed to this rate. Despite efforts to mitigate these barriers through flexible survey methods and extensive outreach, some healthcare workers may have been unable or unwilling to participate.

Participants were selected using a simple random sampling technique from five significant hospitals in central Uganda. The selection of these hospitals was guided by strategic considerations of their regional prominence, their ability to attract healthcare workers from diverse backgrounds, and their accessibility to both urban and rural populations. Each hospital was allotted an equal share of the overall sample, and proportionate sampling was employed for each job category within each hospital. This equal allocation aimed to maintain consistency and comparability across study sites, mitigate potential biases from variations in hospital size, and ensure proportional contributions from each facility to the overall study findings.

### Data collection instruments

Three data collection tools were utilized: a questionnaire covering socio-demographic information, the World Health Organization Quality of Life Scale (WHOQOL-Bref) [23], and The Professional Quality of Life Scale (ProQOL-5)) [24]. The WHOQOL-Bref is a self-administered questionnaire consisting of 26 items. Two items assess general quality of life (items 1 and 2), while the remaining items are divided into four domains: physical, psychological, social, and environmental. Each item is scored on a five-point Likert scale. A higher score indicates a higher quality of life. In this study, the Cronbach’s alpha coefficient for the WHOQOL-Bref was 0.805, suggesting satisfactory internal consistency reliability. The ProQOL-5 was utilized to measure burnout. It is a 30-item self-report questionnaire designed to assess burnout (10 items), compassion fatigue (10 items), and compassion satisfaction (10 items). A burnout score of 22 signifies a low level, 23–41 denotes an average level, and 42 and above indicates a high level of burnout [24]. In the current study, the Cronbach’s alpha coefficient for the ProQOL-5 was 0.89, indicating a high level of internal consistency reliability.

### Procedure

After ethical approval, five research assistants with experience in research were trained on tools for data collection, the purpose of the study, and ethical considerations. After that, we contacted officers of different health facilities (public and private; rural and urban ones) in central Uganda to inform their healthcare workers about our study and request that they collaborate. The participants were informed of the purpose of the study and were encouraged to complete a self-administered questionnaire. There was a trained psychologist in case any participant needed help. It took approximately 23 minutes to complete the survey.

### Data analysis

The data were analyzed using STATA version 17 software at descriptive, bivariate, and multivariate levels of analysis. Descriptive analysis was conducted to report on the general distribution of the sample, presenting the mean and standard deviation for numerical data and frequencies and simple proportions for binary and categorical data. The overall quality of life and the four domains—physical, psychological, social, and environmental—were considered dependent variables, while demographic factors and burnout served as independent variables. The relationship between the dependent and independent variables was assessed through an independent t-test and a one-way analysis of variance (ANOVA) test for binary and categorical independent variables, respectively. The p-value was reported, and relationships were considered significant at p < 0.2. Various assumptions were tested, including normality through the Shapiro-Wilk test. At the multivariable level of analysis, a multiple linear regression analysis was performed using a forward selection method. The level of significance was set at 5% (p < 0.05).

### Ethical considerations

In adherence to ethical standards, our study protocol underwent a thorough review and gained approval from the Lira University Ethics Committee (LUREC 2023–24) (S1 File). This signifies a commitment to protecting the wellbeing, rights, and privacy of the participants. In the process, written informed consent was obtained, ensuring that participants were fully aware of the study’s purpose (S2 File), potential risks, and benefits. Confidentiality measures were implemented to safeguard the anonymity of the participants, and steps were taken to minimize any potential harm.

## Results

### Demographic information of the participants

Table 1 presents the demographic characteristics of the respondents. More than half of the participants, 297 (50.9%), were aged less than 29 years, 340 (62.0%) were female, and 352 (64.0%) had a spouse or partner. Additionally, a majority of the respondents, 242 (44.2%), had worked for less than 5 years, and 255 (46.5%) were nurses.

**Table 1:** Demographic information of the participants.

Variable	Frequency (%)
**Age**	
<29 years	279 (50.9)
29–38 years	193(35.2)
>39 years	76(13.9)
**Gender**	
Female	340(62.0)
Male	208(38.0)
**Marital status**	
With spouse	352(64.2)
Without spouse	196(35.8)
**Work experience**	
<5 years	242(44.2)
5–10 years	140(25.6)
>10 years	166(30.3)

### Quality of life

Table 2 reveals that the mean score for overall quality of life was 10.71 (±4.89). Furthermore, the mean scores varied across domains, ranging from 10.19 (±5.02) in the social domain to 13.58 (±2.15) in the physical domain.

**Table 2:** Quality of life scores.

Quality of Life	Mean	Standard deviation
Overall Quality of Life	10.71	4.89
**Domains**		
Physical	13.58	2.15
Psychosocial	10.68	3.66
Social relationships	10.19	5.02
Environmental	12.14	1.84

### Relationship of demographic characteristics and burnout with quality of life scores

Results in Table 3 show that 218 (39.8%) participants reported a high level of burnout, while only 181 (33.0%) indicated a low level of burnout. Following independent t-tests and one-way analysis of variance for binary and categorical predictor variables, Table 3 indicates associations between various factors and overall quality of life. Specifically, age (p < 0.001), marital status (p = 0.002), work experience (p < 0.001), job category (p < 0.001), supervisor support (p < 0.001), sleep quality (p < 0.001), and burnout (p < 0.001) were found to be associated with overall quality of life. Furthermore, Table 3 demonstrates associations between age, work experience, job category, work load, supervisor support, sleep quality, and burnout with the four domains of quality of life, all with p < 0.05. Additionally, gender (p = 0.037) and marital status (p = 0.001) were only found to be associated with the physical quality of life domain.

**Table 3:** Relationship of demographic factors and burnout with quality of life scores.

Variable	Frequency (%)	Overall Quality of Life	Domain 1	Domain 2	Domain 3	Domain 4
			P value	P value	P value	P value
**Age**						
<29 years	279 (50.9)	<0.001	<0.001	<0.001	<0.001	<0.001
29–38 years	193(35.2)					
>39 years	76(13.9)					
**Gender**						
Female	340(62.0)	0.130	0.037	0.105	0.556	0.706
Male	208(38.0)					
**Marital status**						
With spouse	352(64.2)	0.002	0.001	0.136	0.812	0.343
Without spouse	196(35.8)					
**Work experience**						
<5 years	242(44.2)	<0.001	<0.001	<0.001	0.010	0.001
5–10 years	140(25.6)					
>10 years	166(30.3)					
**Job category**						
Physician	190(34.7)	<0.001	<0.001	<0.001	<0.001	<0.001
Nurse	255(46.5)					
Technician	103(18.8)					
**Weekly load**						
30–38 hours	337(61.5)	0.129	0.135	0.103	0.555	0.703
40 hours and above	211(38.5)					
**Supervisor support**						
Yes	318(58.0)	<0.001	<0.001	<0.001	<0.001	<0.001
No	230(42.0)					
**Workplace violence**						
Yes	217(39.6)	0.130	0.127	0.107	0.555	0.708
No	331(60.4)					
**Quality sleep**						
Yes	312(56.9)	<0.001	<0.001	<0.001	<0.001	0.001
No	236(43.1)					
**Burnout**						
Low	181(33.0)	<0.001	<0.001	<0.001	<0.001	<0.001
Average	149(27.2)					
High	218(39.8)					

Domains (1 = Physical, 2 = Psychological, 3 = Social, 4+Environmental)

### Predictors of overall quality of life scores

Table 4 indicates that job category, sleep quality, and burnout were predictors of overall quality of life scores. Being a nurse reduced the overall quality of life score by 1.22 (β = -1.22; p<0.001) compared to being a physician. Reporting no deprivation of sleep at night was associated with a 3.43 increase in the overall quality of life score (β = 3.45; p<0.001) compared to reporting having quality sleep at night. Finally, having an average level of burnout (β = -2.60; p<0.001) and a high level of burnout (β = -6.02; p<0.001) were associated with a 2.6 reduction and a 6 reduction in the quality of life scores, respectively, compared to a low level of burnout.

**Table 4:** Predictors of overall quality of life.

Quality of Life	β Coefficient	95% confidence interval
**Job category**		
Physician	Ref	
Nurse	-1.22	-1.78, -0.66***
Technician	-0.15	-0.89, 0.60
**Sleep quality**		
Yes	Ref	
No	3.43	2.73, 4.14***
**Burnout**		
Low	Ref	
Average	-2.60	-3.45, -1.75***
High	-6.02	-6.88, -5.15***

*** p<0.001

** p<0.01

* p<0.05

### Predictors of physical, psychological, social and environmental quality of life

Table 5 indicates that being older (39 years and above) was associated with an increase in scores for physical (β = 2.03; p<0.001), psychological (β = 1.57; p<0.01), and social quality of life (β = 3.05; p<0.05) compared to younger age (less than 29 years). Nurses had a reduced physical quality of life score (β = -0.72; p<0.001) compared to physicians. Additionally, technicians had scores reduced for all domains of quality of life: physical (β = -1.57; p<0.001), psychological (β = -1.51; p<0.01), social (β = -4.49; p<0.01), and environmental (β = -2.10; p<0.001) compared to physicians.

**Table 5:** Predictors for physical, psychological, social and environmental quality of life scores.

Predictor	Physical	Psychological	Social relationships	Environmental
	β (95% CI)	β (95% CI)	β (95% CI)	β (95% CI)
**Age**				
<29 years	Ref	Ref	Ref	Ref
29–38 years	-0.09(-0.59, 0.41)	0.18(-0.21, 0.56)	0.70(-0.15, 1.55)	-0.28(-0.58, 0.02)
>39 years	2.03(1.03, 3.03)***	1.57(0.52, 2.61)**	3.05(0.40, 5.71)*	0.66(-0.49, 1.18)
**Job category**				
Physician	Ref	Ref	Ref	Ref
Nurse	-0.72(-1.21, -0.23)**	-0.04(-0.45, 0.38)	-0.89(-1.78, -0.00)	-0.17(-0.48, 0.14)
Technician	-1.57(-2.48, -0.66)***	-1.51(-2.51, -0.50)**	-4.49(-7.02, -1.95)**	-2.10(-3.22, -0.96)***
**Management support**				
Yes	Ref	Ref	Ref	Ref
No	-0.37(-0.67, -0.10)*	0.07(-0.17, 0.31)	-0.16(-0.86, 0.53)	-0.53(-0.72, -0.33)***
**Quality of sleep**				
Yes	Ref	Ref	Ref	Ref
No	0.22(-0.02, 0.46)	0.15(-0.13, 0.45)	-1.11(-1.83, -0.39)**	-0.73(-1.06, -0.39)***
**Burnout**				
Low	Ref	Ref	Ref	Ref
Average	-2.29(-2.71, -1.88)***	-2.18(-2.70, -1.66)***	-4.76(-5.76, -3.77)***	-0.95(-1.39, -0.50)***
High	-2.13(-2.53, -1.73)***	-7.67(-8.14, -7.23)***	-9.60(-10.51, -8.70)***	-3.19(-3.63, -2.75)***

*** p<0.001

** p<0.01

* p<0.05

Healthcare workers who reported that they did not receive support from their supervisors had reduced scores for quality of life in the physical domain (β = -0.37; p<0.05) and environmental domain (β = -0.73; p<0.001) compared to those who received supervisor support. Compared to respondents who reported deprivation of sleep at night, those who did not report the deprivation had an increase in the quality of life scores for the social (β = -1.11; p<0.01) and environmental domains (β = -0.73; p<0.001).

Finally, average and high levels of burnout were associated with an overall reduction in quality of life scores across the four domains (p<0.001) compared to low levels of burnout. For the physical domain, there were comparable reductions in quality of life scores for the average level (β = -2.29; p<0.001) and high level of burnout (β = -2.13; p<0.001). In the psychological, social, and environmental domains, high levels of burnout were associated with a higher reduction in quality of life scores compared to low levels of burnout.

## Discussion

Our study aimed to assess the relationship between burnout and quality of life among healthcare workers in central Uganda. Our investigation revealed that nearly 40% of healthcare workers in central Uganda reported experiencing a high level of burnout. This finding underscores the significant impact of burnout on the quality of life of healthcare workers in the region. It indicates that a considerable proportion of healthcare workers are facing emotional exhaustion, depersonalization, and reduced personal accomplishment, which are characteristic components of burnout syndrome [25]. The wellbeing of healthcare workers in crucial not only for individual health but also for the effectiveness of the healthcare system as a whole. This results result mirror other studies [26]. The mean score for overall quality of life at 10.71 (±4.89) revealed in our study further quantifies the impact of burnout on the well-being of the healthcare workers. This score signifies the aggregated perception of quality of life among the participants, where a higher score indicates a better quality of life and a lower score indicates a poorer quality of life. The relatively low mean scores in this study highlight the challenges these professionals face in various aspects of their lives. To prevent burnout escalation, addressing its root causes and implementing targeted interventions is crucial [22]. Effective strategies like mindfulness-based stress reduction and resilience training can enhance coping skills [27]. Organizational efforts such as workload management and fostering supportive environments are also effective [27].

In our study, the mean scores for quality of life varied across different domains. Specifically, we observed a mean score of 10.19 (±5.02) in the social domain, indicating the perceived quality of social relationships and interactions among participants. On the other hand, in the physical domain, we found a mean score of 13.58 (±2.15), reflecting participants’ assessment of their physical health and well-being. A lower mean score in the social domain may suggest challenges or dissatisfaction with social interactions and support networks among healthcare workers. Conversely, a higher mean score in the physical domain indicates better perceived physical health and functioning among participants. Our results compare favorably with other studies [28, 29]. Recognizing theses variations allows for more targeted approach to intervention strategies, addressing these specific needs of healthcare workers in different aspects of their lives.

A significant negative association between burnout and quality of life scores, with a 6-point reduction in quality of life for healthcare workers with high burnout levels (β = -6.02; p<0.001) highlights the severity of the impact of burnout on overall quality of life of healthcare workers. The substantial reduction in quality of life scores signifies that burnout not only affects one’s professional life but permeates into various dimensions of personal wellbeing. In line with previous studies [15], our results show that burnout not only affects the emotional and mental states of healthcare workers but also has tangible implications for their overall quality of life. The implications of our results could be crucial for designing targeted interventions and support systems to mitigate burnout among healthcare workers in central Uganda, ultimately improving their quality of life and, by extension, the quality of care they provide.

Our findings shed light on specific factors influencing the overall quality of life scores of healthcare workers. One noteworthy result is the impact of professional roles on quality of life, with being a nurse associated with a significant reduction in the overall quality of life score compared to being a physician. On the other hand, the study highlights disparities between different healthcare professions. Nurses, for instance, exhibit a reduced physical quality of life compared to physicians, indicating potential challenges or stressors unique to their role. Similarly, technicians face diminished scores across all quality of life domains–physical, psychological, social, and environmental–in comparison to physicians. The disparity prompts a closer examination of unique challenges faced by nurses in the healthcare setting. Based on our results and previous studies [30], nurses are under various mental and physical pressures depending on their job status. This is attributed to nurses’ workload since they are responsible for taking care of several patients, the repetition of which causes physical and psychological harm, ultimately affecting the quality of life [31]. This result underscores the need for targeted interventions and support systems tailored to the unique challenges faced by nurses in the healthcare setting.

Also, our results further show that sleep quality in determines overall quality of life among healthcare workers. The association between reporting no deprivation of sleep at night and a substantial increase in the overall quality of life score suggests the integral link between physical well-being and life satisfaction in the healthcare workers. Respondents who reported experiencing sleep deprivation at night demonstrate lower scores in the social and environmental domains of quality of life compared to those who did not report such deprivation. This emphasizes the interconnectedness of sleep patterns and the social and environmental aspects of healthcare workers’ well-being. The heavy workload, poor pay, poor transport system, and poor housing conditions in the Ugandan health system [19] can explain this situation. Evidence from previous studies supports the idea that insufficient sleep is negatively associated with healthcare workers’ wellbeing [32]. This result emphasizes the importance of addressing sleep-related issues as a potential avenue for enhancing the overall quality of life among healthcare workers. As sleep is a fundamental aspect of overall health, interventions aimed at improving sleep quality among healthcare workers may have broader implications for their holistic well-being. Interventions that focus on promoting healthy sleep habits, managing work-related stressors affecting sleep, and creating conducive environments for adequate rest may prove beneficial in positively impacting the well-being of healthcare workers in central Uganda.

0ur findings reveal that older healthcare workers, specifically those aged 39 years and above, experience higher scores in physical, psychological, and social quality of life compared to their younger counterparts (below 29 years). This suggests that as healthcare workers age, there is a positive association with various aspects of their well-being. On the other hand, the study highlights disparities between different healthcare professions. Also, our findings show that healthcare workers who reported a lack of support from their supervisors exhibited reduced scores in both the physical and environmental domains of quality of life. This suggests that the presence of supportive leadership plays a pivotal role in influencing the well-being of healthcare workers, particularly in terms of their physical health and the overall work environment. Various studies suggest that supervisor support is linked to health in general [33] and positively associated with physical and mental health [34, 35]. These findings underscore the importance of implementing targeted support strategies for specific age groups and professions could contribute to a more resilient and satisfied healthcare workforce. Also, the findings underscore the importance of organizational support structures and management practices in mitigating burnout and fostering a positive work environment for healthcare workers.

### Strength and limitations of the study

The present study employs a cross-sectional design and utilizes validated tools to comprehensively assess burnout and quality of life among healthcare workers. Secondly, the inclusion of healthcare workers from various settings, including public and private hospitals, enhances the generalizability of our findings. Lastly, our findings offer practical insights for designing targeted interventions to address burnout and improve the quality of life for healthcare workers in central Uganda. However, our study had its limitations: the cross-sectional nature of our study limits its ability to establish causal relationships between variables. A longitudinal study could provide deeper insights into the development of burnout among healthcare workers over time. By observing changes and causal relationships more effectively, longitudinal studies offer a clearer understanding of how burnout evolves and its long-term impacts. Secondly, the reliance on self-reported data may introduce response bias, and participants may underreport or overreport certain factors. Also, while the study includes diverse healthcare settings, the findings may not be fully generalizable to all healthcare workers in central Uganda or other regions.

## Conclusion

The findings highlight the interconnectedness of socio-demographic factors, burnout, and specific aspects of quality of life. Notably, job category, supervisory support, sleep quality, and burnout emerge as influential factors shaping healthcare workers’ wellbeing. Nurses and technicians face unique challenges, indicating the necessity of tailored interventions. Addressing insufficient supervisory support, burnout, and sleep-related issues is identified as a potential avenue for enhancing the overall quality of life among healthcare workers. Overall, our study provides a valuable foundation for future longitudinal studies and interventions development to enhance the wellbeing of healthcare workers in resource-limited settings.

## Supporting information

S1 File Lira University Ethics Committee clearance (LUREC 2023–24). (PDF)

S2 File Human participants research checklist. (DOCX)

S3 File (SAV)
