# Supplementary material for: Burnout and quality of life among healthcare workers in central Uganda
Source: PLoS One. 2024 Aug 19;19(8):e0305713. doi: 10.1371/journal.pone.0305713 (PMC11332927; doi:10.1371/journal.pone.0305713)
Supplement: S1 File — (PDF) [file pone.0305713.s001.pdf]

LIRA UNIVERSITY

P.O. Box 1035
Lira, Uganda
Tel: +256 414-694716
Web: www.lirauni.ac.ug
Email: lirarec@lirauni.ac.ug.

RESEARCH ETHICS COMMITTEE

05/04/2023

To: AMIR KABUNGA

LIRA UNIVERSITY
0777929576

Type: Initial Review

Re: LUREC 2023-24: BURNOUT AND QUALITY OF LIFE AMONG HEALTHCARE WORKERS IN CENTRAL UGANDA, 3.0, 2023-04-05

I am pleased to inform you that at the **24th** convened meeting on **05/04/2023**, the lira University REC, committee meeting, etc voted to approve the above referenced application.

Approval of the research is for the period of **05/04/2023** to **08/02/2024.**

As Principal Investigator of the research, you are responsible for fulfilling the following requirements of approval:

1. All co-investigators must be kept informed of the status of the research.
2. Changes, amendments, and addenda to the protocol or the consent form must be submitted to the REC for rereview and approval **prior** to the activation of the changes.
3. Reports of unanticipated problems involving risks to participants or any new information which could change the risk benefit: ratio must be submitted to the REC.
4. Only approved consent forms are to be used in the enrollment of participants. All consent forms signed by participants and/or witnesses should be retained on file. The REC may conduct audits of all study records, and consent documentation may be part of such audits.
5. Continuing review application must be submitted to the REC **eight weeks** prior to the expiration date of **05/04/2023**
   in order to continue the study beyond the approved period. Failure to submit a continuing review application in a timely fashion may result in suspension or termination of the study.
6. The REC application number assigned to the research should be cited in any correspondence with the REC of record.
7. You are required to register the research protocol with the Uganda National Council for Science and Technology (UNCST) for final clearance to undertake the study in Uganda.

The following is the list of all documents approved in this application by Lira University REC:

No.	Document Title	Language	Version Number	Version Date
1	Protocol	English	3.0	2023-04-05
2	Data collection tools	ENGLISH	3.0	2023-04-05
3	Informed Consent forms	LUO	3.0	2023-04-05
4	Informed Consent forms	ENGLISH	3.0	2023-04-05
5	covid 19 risk management plan	ENGLISH	3.0	2023-04-05

Yours Sincerely

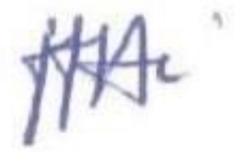

Dr. Gerald OBAI
For: Lira University REC

Powered by TCPDF (www.tcpdf.org)
